# Psychopathological profile of patients with chronic low back pain

**DOI:** 10.1192/j.eurpsy.2023.2049

**Published:** 2023-07-19

**Authors:** I. Martin-Villalba, P. Gavín

**Affiliations:** Hospital Clínic i Provincial de Barcelona, Barcelona, Spain

## Abstract

**Introduction:**

Chronic back pain is one of the leading causes of disability and treatment is often ineffective. Approximately 85% of cases are primary, for which the peripheral etiology cannot be identified, and maintenance factors include fear, avoidance, and beliefs that pain indicates injury.Studies suggest that an excessively negative orientation towards pain (pain catastrophism) and fear of movement/(re)injury (kinesiophobia) are important in the aetiology of chronic low back pain and associated disability.

**Objectives:**

The aim is to identify the psychopatological profile (including levels of movement phobia and catastrophic thinking) in patients with chronic low back pain who participate in a multidisciplinary group Comprehensive Care Program for Patients with Chronic Pain (PAINDOC) in the Pain Unit of the Hospital Clínic i Provincial de Barcelona.

**Methods:**

A cross-sectional descriptive study of 75 patients aged between 28 and 68 years who participate in the treatment is carried out. A clinical interview was carried out, anxiety and depression symptoms were assessed with thHospital Anxiety and Depression Scale (HADS), Kinesiophobia was evaluated with the Tampa Scale Kinesiophobia (TSK-11SV) and catastrophism with the Pain Catastrophizing Scale (PCS).

**Results:**

The results show that the patients presented a high level of catastrophic thinking, especially in the subgroup of women with a longer duration of pain.Movement phobia was observed in 27% of patients, and correlated with feelings of depression and anxiety.

**Conclusions:**

Catastrophism is a multidimensional psychological construct that encompasses elements such as rumination, magnification, and incapacity.Catastrophic thinking in relation to pain is a risk factor for chronic pain. Kinesiophobia is based on the fear avoidance model and perpetuates a cycle of heightened fear of pain, disability, and depression. These constructs can be very useful to evaluate in patients to reduce the intensity of pain, the associated emotional suffering and reduce the probability that the pain will become chronic.

**Disclosure of Interest:**

None Declared

